# Oil of Cinnamon

**Published:** 1898-02

**Authors:** 


### Oil of Cinnamon.

Inhaling of this causes diminution in number and disappearance of tubercle bacilli from the sputum in phthisis, and tends to cure the disease by arresting growth of bacilli and by preventing organisms capable of growth from passing along the bronchi to infect new lobules.—*Thompson.*

